# Minimally invasive surgery vs laparotomy for early stage cervical cancer: A propensity score‐matched cohort study

**DOI:** 10.1002/cam4.3527

**Published:** 2020-11-25

**Authors:** Danian Dai, He Huang, Yanling Feng, Ting Wan, Zhimin Liu, Chongjie Tong, Jihong Liu

**Affiliations:** ^1^ Department of Gynecologic Oncology State Key Laboratory of Oncology in South China Collaborative Innovation Center for Cancer Medicine Sun Yat‐sen University Cancer Center Guangzhou Guangdong China; ^2^ Department of Gynecology and Obstetrics The Fifth Affiliated Hospital of Sun Yat‐Sen University Zhuhai Guangdong China

**Keywords:** cohort study, early stage cervical cancer, laparotomy, minimally invasive surgery, propensity score matching

## Abstract

**Objective:**

To compare the long‐term oncologic outcomes of minimally invasive surgery (MIS) vs laparotomy for patients with stage IB (2018 FIGO) cervical cancer.

**Methods:**

A matched retrospective study of cervical cancer patients who underwent MIS or laparotomy at Sun Yat‐sen University Cancer Center from January 2012 to December 2015 was carried out. Patients were restaged according to the 2018 FIGO staging system for cervical cancer, 700 cases with stage IB cervical cancer were enrolled. Propensity score matching (PSM) was performed by software SPSS version 22.0, and a total of 426 patients were enrolled and analyzed. Oncologic outcomes were compared between patients undergoing MIS vs laparotomy.

**Results:**

After PSM, there were no statistical differences in other baseline characteristics between MIS and laparotomy, except for age (*p* = 0.008). In all stage IB patients, MIS group had significantly lower disease‐free survival (DFS) rate and overall survival (OS) rate compared with laparotomy group (5‐year DFS rate, 87.5% vs 94.1%, hazard ratio for disease recurrence, 2.403; 95% CI, 1.216‐4.744; 5‐year OS rate, 92.3% vs 98.1%, hazard ratio for death, 3.719; 95% CI, 1.370‐10.093). In stage IB1 patients population, MIS was still associated with worse DFS and OS compared to laparotomy (5‐year DFS rate: 89.5% vs 100%, *p* = 0.012; 5‐year OS rate: 93.4% vs 100%, *p* = 0.043). Even in stage IB1 patients without lymph vascular space invasion, worse oncologic outcome could be observed in MIS group (DFS: *p* = 0.021; OS: *p* = 0.076).

**Conclusion:**

Our study suggested that laparotomy resulted in better OS and DFS compared with MIS among patients with stage IB cervical cancer. Even in stage IB1 patients without lymph vascular space invasion (2018 FIGO), laparotomy might be still an oncologically safer approach.

## Graphical abstract

Recent studies have shown that patients with early‐stage cervical cancer who underwent minimally invasive surgery (MIS) had a worse prognosis than that of laparotomy; However, whether minimally invasive radical hysterectomy is available for relatively low‐risk early patients under the 2018 FIGO guidelines have yet to be studied. A propensity score‐matched cohort study examining patients with stage IB (2018 FIGO) cervical cancer of relatively low‐risk who underwent radical hysterectomy found that the five‐year overall survival and disease‐free survival of MIS respectively reduced by 5.8% and 6.6% compared with laparotomy. The surgical approaches do have a significant difference for survival of patients with early cervical cancer who underwent radical hysterectomy. Even in stage IB1 patients with low‐risk, the oncologic outcomes of laparotomy is better compared with MIS.

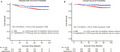

## 1 INTRODUCTION

Cervical cancer ranks fourth in the incidence and mortality of the most common tumors in women, and there are 570,000 new cases of cervical cancer all over the world in 2018, and 310,000 patients died in the same period.^1^ Conventional abdominal radical hysterectomy plus lymph node dissection (LND) was considered as the mainstream operation for treating patients with early stage cervical cancer.^2^, ^3^ Since the first case of cervical laparoscopic surgery reported internationally in 1992,^4^ a large number of retrospective studies of laparoscopic and open radical resection for early cervical cancer treatment showed no significant difference in long‐term oncologic outcome.^5^, ^6^, ^7^, ^8^, ^9^ Also, the previous National Comprehensive Cancer Network (NCCN) and International Federation of Gynecology and Obstetrics (FIGO) also have recognized the feasibility of laparoscopic surgery in their guidelines.^10^, ^11^ Therefore, laparoscopic surgery has been widely used in the treatment of early cervical cancer because of its advantages, such as less intraoperative blood loss, shorter hospital stay, and minimally invasive than open surgery.^12^, ^13^ However, a retrospective study and a multicenter prospective randomized controlled trial (Laparoscopic Approach to Cervical Cancer, LACC) published in 2018 indicated, compared with open surgery, the disease‐free survival (DFS) and overall survival (OS) of patients with early stage cervical cancer who underwent minimally invasive surgery (MIS) were significantly lower.^14^, ^15^ Unlike developed countries with good cervical cancer screening and HPV vaccination coverage, the incidence of cervical cancer in China is still on the rise. China's cancer statistics in 2016 showed that from 2000 to 2007 and from 2007 to 2011, the annual percentage changes of cervical cancer in China were 15.6% and 4.1%, respectively.^16^ It can be seen that although the incidence of cervical cancer in China has slowed down, it still maintains an upward trend. By far, limited data are comparing the outcome of stage IB patients undergoing surgical treatment through different surgical approaches based on the 2018 international federation of gynecology and obstetrics (FIGO) staging. Our institute is the largest cancer center in South China, and the goal of this study is to compare the long‐term oncologic outcomes in relatively low‐risk cervical cancer patients of stage IB (2018 FIGO) undergoing MIS vs laparotomy in our single institute.

## 2 PATIENTS AND METHODS

### 2.1 Patients and data sources

We retrospectively collected the clinical and pathological data of 700 stage IB1 (2009 FIGO) patients who underwent abdominal or laparoscopic radical hysterectomy ±LND in Sun Yat‐sen University Cancer Center (SYSUCC) from January, 2012 to December, 2015. Then, these patients were restaged according to 2018 FIGO^17^ and MIS includes only laparoscopic surgery and of the 700 patients, 286 underwent MIS and 414 underwent laparotomy. About 560 patients were included in the analysis according to the following inclusion. Inclusion criteria: (1) Invasive cervical cancer diagnosed by pathology (including squamous cell carcinoma, adenocarcinoma, and adenosquamous carcinoma); (2) Patients initially treated in SYSUCC; (3) patients died of cervical cancer. Exclusion criteria: (1) diagnosed with a second tumor; (2) postoperative pathology reported lymph node metastasis; (3) patients with neoadjuvant chemotherapy; (4) patients with parametrial involvement; (5) patients with rare histological types. This study was approved by the ethics committee of Sun Yat‐sen University Cancer Center.

### 2.2 Clinical management and follow‐up

All patients underwent radical hysterectomy (type II or III) with pelvic and/or para‐aortic lymphadenectomy. Patients with two or more intermediate risk factors [lymph vascular space invasion (LVSI), >1/2 stromal invasion or tumor size >4 cm] received radiotherapy ±chemotherapy. All operations require senior attending doctors or doctors with senior titles to perform. All patients were followed up with physical examination and/or radiographic imaging every 3 months for 2 years after surgery, then, every 6 months for the 3rd to 5th years and yearly thereafter. All patients’ survival statuses were confirmed in Jun. 2020, and median follow‐up was 68.7 months (range, 8.6‐99.1 months) for all patients.

### 2.3 Observation indexes and definitions

(1) Recurrence and survival: Recurrence site was classified as intrapelvic or extra‐pelvic. If a patient had both intrapelvic recurrence and extra‐pelvic metastasis, the patient was classified as extra‐pelvic metastasis. Disease‐free survival (DFS) was defined as from the date of surgery to the date of relapse, metastasis, or last follow‐up. Overall survival (OS) was computed from the date of surgery to the date of death or last follow‐up. (2) Operation‐related indicators: including operative time, estimated blood loss, and length of hospital stay. The length of hospital stay was counted from the first postoperative day.

### 2.4 Propensity score matching analysis

In this study, propensity score matching (PSM) was used to reduce bias due to an imbalance in observed variables between MIS and laparotomy groups. Four factors of baseline characteristics (age, radiotherapy, pathologic grade, and FIGO staging) were selected as covariates in PSM model and match tolerance set to 0.01. Propensity scores of individuals were calculated with logistic regression analysis (SPSS version 22.0, Chicago, IL), then, the optimal 1:1 matching between MIS and laparotomy patients was produced based on propensity scores. After matching, the distribution of the rest observed variables was similar in MIS and laparotomy groups, except age.

### 2.5 Statistical analysis

Statistical analysis was performed with SPSS 22.0 software (SPSS version 22.0, Chicago, IL). Normality testing (D’Agostino and Pearson test) was performed to evaluate whether data were sampled from a Gaussian distribution. After PSM, we assessed the balance of baseline characteristics of the two groups with independent‐samples *t* tests or Wilcoxon rank‐sum test for continuous variables and chi‐square test for categorical variables. The Kaplan‐Meier method was used to estimate OS and DFS (log‐rank test) and the Cox multivariate proportional hazards regression model was used to determine the independent factors that influence survival and recurrence based on the observed variables. *p* < 0.05 was considered statistically significant.

## 3 RESULTS

### 3.1 Baseline characteristics before and after PSM

**Figure 1:**
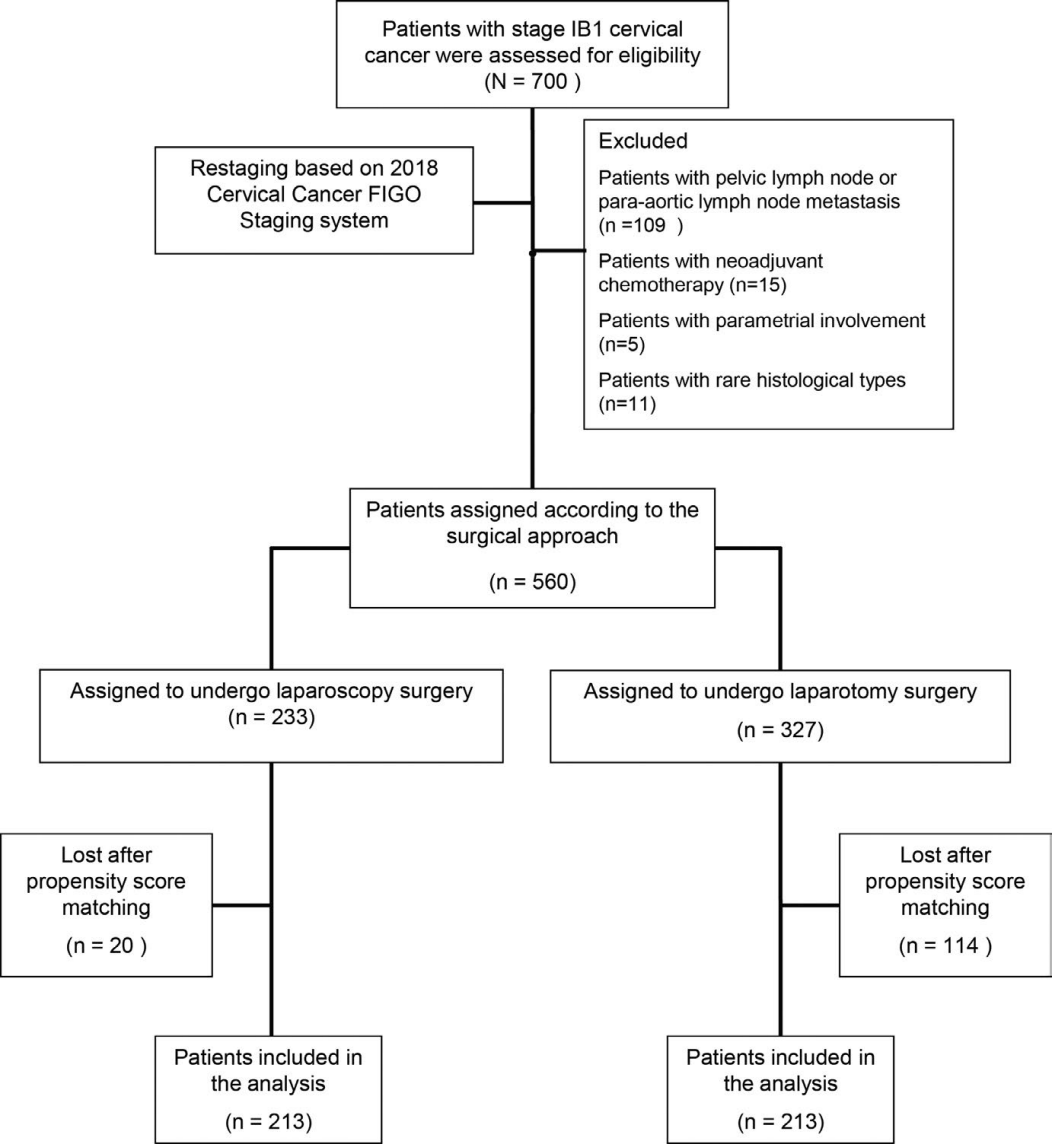
CONSORT diagram

A total of 700 patients with stage IB1 cervical cancer (2009 FIGO^18^) were collected; and eventually, 560 patients were included in the analysis after restaging based on the 2018 FIGO staging system (Figure 1). Within this cohort, 233 underwent MIS and 327 underwent laparotomy. The baseline clinical pathological characteristics are listed in Table 1. In comparison with the MIS group, the age was older (*p* < 0.001) and FIGO staging was worse (*p* = 0.012) in the laparotomy group. In addition, MIS group had less proportion of radiotherapy and more patients with well pathologic grade compared with the laparotomy group (all *p* < 0.05). After PSM, 426 patients (MIS = 213; Laparotomy = 213) were matched, and Table 1 shows the patients’ characteristics. The average age of patients in MIS group was significantly smaller than that of patients in the laparotomy group (*p* = 0.008). No other significant differences between MIS and laparotomy groups were observed in terms of either BMI, chemotherapy, radiotherapy, depth of cervical stromal invasion (DCSI), histology, pathological grade, resection margin, LVSI, or FIGO staging.

**Table 1:** Baseline characteristics before and after propensity score matching

Variables	Before PSM			After PSM		
	MIS (n = 233)	Laparotomy (n = 327)	*p* value	MIS (n = 213)	laparotomy (n = 213)	*p* value
Age (year, Mean ± SD)^a^	44.9 ± 8.4	48.0 ± 9.2	**<0.001** *	45.2 ± 8.3	47.3 ± 8.6	**0.008** *
BMI (kg/m^2^, Mean ± SD)^a^	23.0 ± 3.4	23.2 ± 3.4	0.419	23.0 ± 3.1	23.2 ± 3.4	0.377
ACT^b^			0.587			1.000
No	166 (71.2%)	226 (69.1%)		156 (73.2%)	156 (73.2%)	
Yes	67 (28.8%)	101 (30.9%)		57 (26.8%)	57 (26.8%)	
Radiotherapy^b^			**0.002** *			0.306
No	152 (65.2%)	170 (52.0%)		136 (63.8%)	146 (68.5%)	
Yes	81 (34.8%)	157 (48.0%)		77 (36.2%)	67 (31.5%)	
DCSI^b^			0.321			0.130
Depth <5 mm	47 (20.2%)	50 (15.3%)		42 (19.7%)	46 (21.6%)	
Shallow muscularis (<1/2)	84 (36.1%)	124 (37.9%)		78 (36.6%)	94 (44.1%)	
Deep muscularis (≥1/2)	102 (43.8%)	153 (46.8%)		93 (43.7%)	73 (34.3%)	
Histology^b^			0.234			0.165
Squamous	177 (76.0%)	240 (73.4%)		161 (75.6%)	146 (68.5%)	
Adenocarcinoma	52 (22.3%)	73 (22.3%)		48 (22.5%)	58 (27.2%)	
Adenosquamous	4 (1.7%)	14 (4.3%)		4 (1.9%)	9 (4.2%)	
Pathologic grade^b^			**<0.001** *			0.425
G1	27 (11.6%)	10 (3.1%)		16 (7.5%)	10 (4.7%)	
G2	101 (43.3%)	138 (42.2%)		96 (45.1%)	94 (44.1%)	
G3	105 (45.1%)	179 (54.7%)		101 (47.4%)	109 (51.2%)	
Resection margin^b^			0.563			0.401
Negative	219 (94.0%)	311 (95.1%)		199 (93.4%)	203 (95.3%)	
Positive	14 (6.0%)	16 (4.9%)		14 (6.6%)	10 (4.7%)	
LVSI^b^			0.841			0.184
Negative	165 (70.8%)	229 (70.0%)		152 (71.4%)	164 (77.0%)	
Positive	68 (29.2%)	98 (30.0%)		61 (28.6%)	49 (23.0%)	
FIGO staging^b^			**0.012** *			1.000
Ib1	61 (26.2%)	55 (16.8%)		51 (23.9%)	51 (23.9%)	
Ib2	139 (59.7%)	206 (63.0%)		130 (61.0%)	130 (61.0%)	
Ib3	33 (14.2%)	66 (20.2%)		32 (15.0%)	32 (15.0%)	

Abbreviations: ACT, adjuvant chemotherapy; DCSI, depth of cervical stromal invasion; FIGO, International Federation of Gynecology and Obstetrics; G1, well differentiated; G2, moderately differentiated; G3, poorly differentiated; LVSI, lymph vascular space invasion; MIS, minimally invasive surgery.

^a^ Using *t* test or ANOVA, *p* < 0.05 was considered statistically significant.

^b^ Using Chi‐squared test, *p* < 0.05 was considered statistically significant.

* *p* < 0.05, statistically significant.

### 3.2 Operation‐related characteristics

Patients in the MIS group had an average operative time of 247.7 minutes, while it was 212.0 minutes in the laparotomy group. This difference was statistically significant (*p* < 0.001). The mean estimated blood loss (EBL) was 140.0 ml in the MIS group and 199.1 ml in laparotomy (*p* < 0.001). A significant shorter mean hospital stay was recorded in patients who underwent MIS 6.4 days vs 8.0 days in laparotomy, (*p* < 0.001) (Supplementary Table S1).

### 3.3 Recurrence and survival

**Figure 2:**
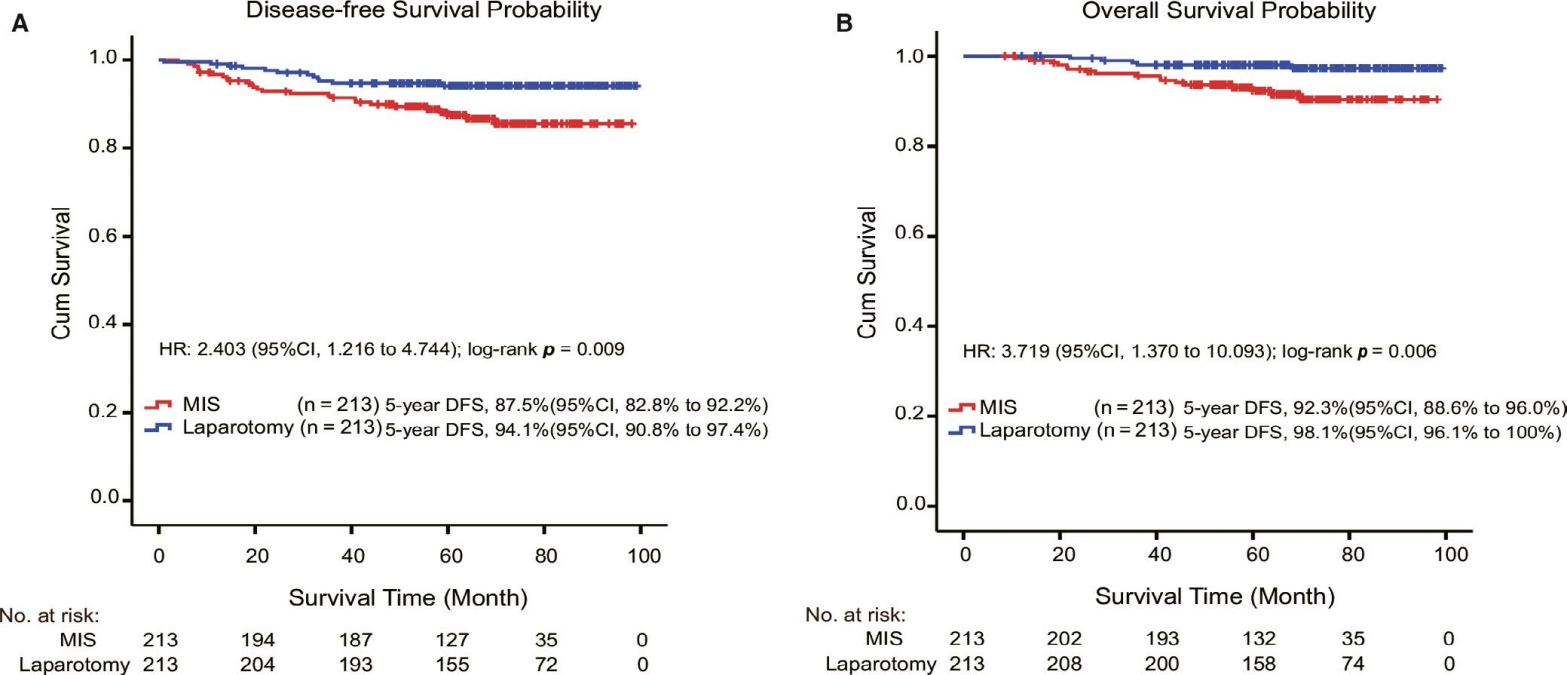
Kaplan‐Meier survival curves for MIS and laparotomy groups. (A) The overall survival (OS) rate of MIS and laparotomy groups for all matched patients. (B) The disease‐free survival (DFS) rate of MIS and laparotomy groups for all matched patients

Median follow‐up was 65.2 months (range, 8.6‐98.2 months) and 71.9 months (range, 12.0‐99.1 months) for MIS and laparotomy group, respectively. During the follow‐up period, disease relapsed in 27 patients in MIS group, and the recurrence ratio was 12.7% (27/213), of which six patients had extra‐pelvic metastasis and 21 had intrapelvic recurrence. However, there were 12 patients experienced recurrence in the laparotomy group, and the recurrence ratio was 5.6% (12/213), of which two patients had extra‐pelvic metastasis and 10 had an intrapelvic recurrence (Supplementary Table S2). Furthermore, 17 patients (8.0%, 17/213) died in MIS group while five patients (2.3%, 5/213) died in laparotomy group (Supplementary Table S1). The 5‐year DFS and OS rates were 87.5% and 92.3% for MIS group and 94.1% and 98.1% for laparotomy group, respectively. For all matched patients, MIS was associated with higher risk of recurrence (HR, 2.403; 95%CI, 1.216‐4.744; log‐rank *p* = 0.009) and disease‐specific death (HR, 3.719; 95%CI, 1.370‐10.093; log‐rank *p* = 0.006) (Figure 2A,B).

### 3.4 Cox logistic regression for prognostic factors

Univariate Cox proportional hazard regression analysis revealed that surgical approach, histology, pathological grade, DCSI, LVSI were potentially predictive factors of prognosis for OS or DFS in patients with stage IB1‐IB3 cervical cancer (all *p* < 0.1) (Table 2). Furthermore, the multivariate survival analysis model revealed the independent predictors of OS and DFS were surgical approach (OS, HR: 3.389; 95% CI: 1.234‐9.305; *p* = 0.018; DFS, HR: 2.023; 95% CI: 1.019‐4.017; *p* = 0.044), and DCSI (OS, HR: 3.671; 95% CI: 1.298‐10.385; *p* = 0.014; DFS, HR: 2.952; 95% CI: 1.447‐6.025; *p* = 0.003) (Table 3).

**Table 2:** Univariate COX regression analysis for overall survival and disease‐free survival in patients with stage Ib1‐Ib3 cervical cancer

Variables	Case	Overall Survival				Disease‐free Survival			
		Survival rate at 5 year (%)^a^	HR	(95%CI)	*p* value	Survival rate at 5 year(%)^a^	HR	(95%CI)	*p* value
Surgical approach									
Laparotomy	213	98.1%	Reference		0.010*	94.1%	Reference		0.012*
MIS	213	92.3%	3.719	1.370‐10.093		87.5%	2.403	1.216‐4.744	
FIGO staging									
Ib1	102	96.6%	Reference		0.765	94.7%	Reference		0.316
Ib2	260	94.8%	1.504	0.499‐4.533		90.3%	1.673	0.686‐4.078	
Ib3	64	94.6%	1.297	0.290‐5.798		86.8%	2.261	0.784‐6.518	
Histology									
Squamous	307	94.8%	Reference		0.098*	91.4%	Reference		0.724
Adenocarcinoma	106	98.0%	0.320	0.074‐1.380		90.1%	1.095	0.530‐2.263	
Adenosquamous	13	84.6%	2.757	0.639‐11.888		84.6%	1.786	0.425‐7.514	
Pathological grade									
G1	26	95.8%	Reference		0.096*	87.8%	Reference		0.183
G2	190	98.4%	0.653	0.076‐5.594		93.8%	0.492	0.139‐1.745	
G3	210	92.3%	1.945	0.258‐14.673		88.5%	0.923	0.278‐3.066	
ACT									
No	312	96.2%	Reference		0.110	90.6%	Reference		0.920
Yes	114	92.4%	0.707	0.462‐1.082		91.5%	1.019	0.711‐1.459	
Radiotherapy									
No	282	95.5%	Reference		0.805	91.8%	Reference		0.325
Yes	144	94.6%	0.947	0.613‐1.462		89.1%	0.859	0.625‐1.182	
Resection margin									
Negative	402	95.7%	Reference		0.017*	91.3%	Reference		0.056*
Positive	24	87.5%	3.750	1.269‐11.088		83.3%	2.502	0.978‐6.397	
DCSI									
Depth <5 mm/shallow muscularis (<1/2)	260	98.0%	Reference		0.001*	95.3%	Reference		<0.001*
Deep muscularis (≥1/2)	166	90.8%	5.633	2.078‐15.273		83.8%	3.712	1.880‐7.328	
LVSI									
Negative	316	96.9%	Reference		0.007*	93.0%	Reference		0.007*
Positive	110	90.4%	3.177	1.375‐7.341		84.8%	2.403	1.275‐4.529	

*p* < 0.1, variables were included in multivariate analysis.

Survival rate at 5 year was calculated based on the Kaplan‐Meier method.

Abbreviations: 95% CI, 95% confidential interval; ACT, adjuvant chemotherapy; DCSI, depth of cervical stromal invasion; FIGO, International Federation of Gynecology and Obstetrics; G1, well differentiated; G2, moderately differentiated; G3, poorly differentiated; HR, hazard ratio; LVSI, lymph vascular space invasion; MIS, minimally invasive surgery.

* *p* < 0.1 was included in the multivariate analysis.

**Table 3:** Multivariate COX regression analysis for overall survival and disease‐free survival in patients with stage Ib1‐Ib3 cervical cancer.

Variables	Overall Survival			Disease‐free Survival		
	HR	(95%CI)	*p* value	HR	(95%CI)	*p* value
Surgical approach			0.018*			0.044*
Laparotomy	Reference			Reference		
MIS	3.389	1.234‐9.305		2.023	1.019‐4.017	
DCSI						
Depth <5 mm/shallow muscularis (<1/2)	Reference		0.014*	Reference		0.003*
Deep muscularis (≥1/2)	3.671	1.298‐10.385		2.952	1.447‐6.025	

Abbreviations: 95% CI, 95% confidential interval; DCSI, depth of cervical stromal invasion; HR, hazard ratio; MIS, minimally invasive surgery.

* *p* < 0.05, statistically significant.

### 3.5 Subgroup analysis

**Figure 3:**
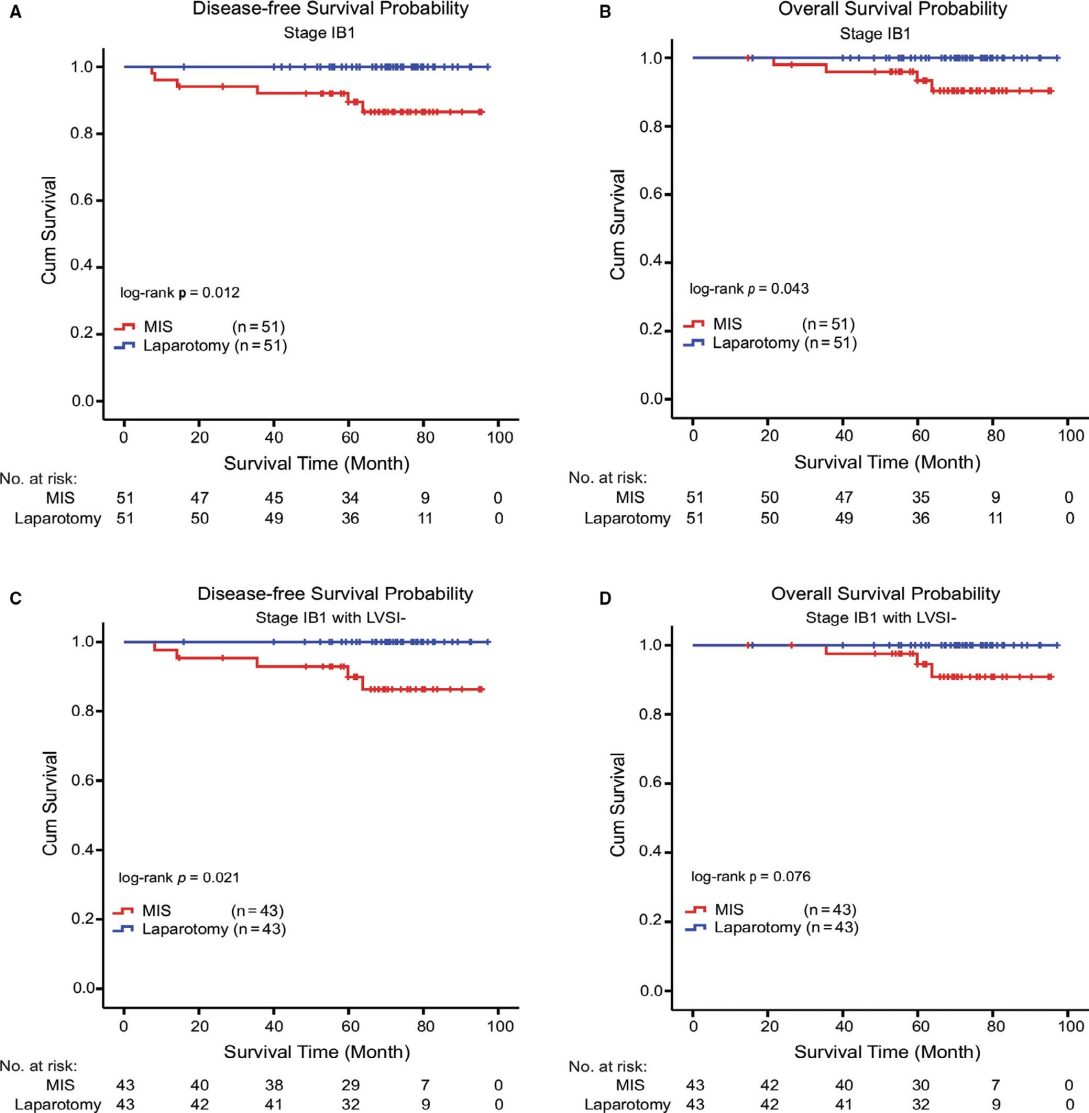
Kaplan‐Meier survival curves stratified by staging IB1 and staging IB1 with LVSI‐. (A and B) The overall survival (OS) rates and disease‐free survival (DFS) rates of different groups in patients with stage IB1. (C and D) The overall survival (OS) rates and disease‐free survival (DFS) rates of different groups in stage IB1 patients with LVSI‐

To explore the survival impact of surgical approach in low‐risk patients (IB1, IB1 with LVSI‐), the DFS and OS were analyzed by Kaplan‐Meier curve between MIS and laparotomy groups (Figure 3). For stage IB1 patients, six cases of recurrence occurred in the MIS group [5‐year DFS, 89.5% (95%CI, 82.1%‐96.9%)], and of which four died [5‐year OS, 93.4% (95%CI, 86.1%‐100%)] (Figure 3A,B). Even in cervical cancer patients of stage IB1 with LVSI‐, five cases of recurrence occurred in the MIS group [5‐year DFS, 89.9% (95%CI, 80.5%‐99.3%)], and of which three died [5‐year OS, 94.5% (95%CI, 87.1%‐100%)] (Figure 3C,D). No cases of recurrence and death were observed in stage IB1 patients of the laparotomy group. The results showed that even in low‐risk patients, MIS group had significantly worse DFS and OS than the laparotomy group.

## 4 DISCUSSION

In this retrospective study, early stage cervical cancer patients (2018 FIGO stage IB1‐IB3) who underwent laparoscopic surgery had lower rates of DFS and OS than that of open approach. Moreover, even for stage IB1 patients with LVSI‐, the minimally invasive surgery was also associated with increased risk of recurrence.

A large retrospective cohort study, in 2018, revealed the shorter DFS and OS outcome of minimally invasive surgery for early stage cervical cancer compared with open surgery.^14^ Among the 2461 patients with early stage cervical cancer (IA2 and IB1, 2009 FIGO stage), the risk of death in the minimally invasive group (1225 patients) was 65% (HR =1.65; 95%CI: 1.22‐2.22; *p* = 0.002) higher than that in the open group (1236 patients). The results of prospective randomized controlled LACC study^15^ showed that the risk of all‐cause death in the minimally invasive group was six times higher than that in the open group (95%CI: 1.77‐20.30), and cervical cancer mortality (4.4% vs 0.6%, HR = 6.56, 95%CI: 1.48‐29.00) and the local recurrence rate (3 years without local recurrence survival rate: 94.3% vs 98.3%, HR = 4.26, 95%CI: 1.44‐12.60) of minimally invasive group was significantly higher compared with open group. Similarly, our data showed that both the 5‐year DFS and OS rates in the MIS group were both worse than those of laparotomy group (Figure 2A,B). Consistent with the previous literatures, our findings also support the superiority of open surgery in the benefit of DFS and OS.

However, the results of LACC study cannot be directly extended to patients with “low‐risk” cervical cancer, that is, IB tumor size <2 cm; no lymphovascular invasion; no lymph node involvement. Therefore, it is still unknown whether minimally invasive surgery could be considered for the abovementioned early stage low‐risk patients. This study sought to provide more information on prognosis of laparoscopy for early stage patients with cervical cancer.

Furthermore, in our study, the stratified survival analysis showed that laparotomy offered significantly better DFS and OS in patients with stage IB1 (2018 FIGO) (Figure 3A,B). It is worth noting that the prognostic benefit of DFS for laparotomy group is more obvious than that of MIS group for stage IB1 patients with LVSI‐ (*p* = 0.021), though OS had no significant differences (*p* = 0.076). The KGOG 1028 study^19^ also showed that for patients with tumors <2 cm, laparoscopic surgery had worse DFS than laparotomy, but there was no difference in OS. Similarly, Alexander et al.^14^ also reported similar results as described above, which published in the New England Journal of Medicine. Recently, Brandt et al. reported that MIS radical hysterectomy did not confer worse oncologic outcomes compared with open surgery.^20^ Notably, in that study, the minimally invasive group had a larger proportion of residual lesions, and a lower proportion of postoperative risk factors. Under this uneven risk factors, it seems that further evidence is needed to conclude that minimally invasive surgery did not bring worse prognosis. In our study, surgical approach was considered as an independent prognostic factors for DFS and OS. Thus, we still need to carefully choose the laparoscopic surgical approach for stage IB1 patients with LVSI‐ (2018 FIGO) and also need more cases to verify our results. Our results also suggest that the safety of MIS may still not be as good as laparotomy for patients with low‐risk early stage cervical cancer.

Considering the clinical risks associated with MIS, FIGO also recommended that open surgery should be preferred for the treatment of early stage cervical cancer patients, including those with stage IA1 with LVSI and stage IB1 cancers.^21^ Our study also provided evidence from the real world to advocate a more cautious application of MIS. However, whether the improvement of MIS operation technology has improved the prognosis of early stage patients and whether it could be applied to patients with low‐risk is still worthy of further study.

The strengths of our research lie in the fact that this is the first clinical retrospective study in the Chinese population based on the 2018 FIGO staging and stratified analysis of early stage patients with low‐risk. Besides, our institution has centralized pathological diagnosis, standardized preoperative evaluation and surgical selection, and unified follow‐up management. More importantly, our study provided evidence for the question of whether stage IB1 or IB1 patients with LVSI‐ could be treated by laparoscope. We do recognize that our study also has the limitations of being a retrospective study. First, the heterogeneity differences between treatment groups still exist, while observed confounders were well balanced after propensity score matching, confounding by unobserved factors remains a possibility. We believe that although the MIS group has an advantage in age, it does in turn confirm the advantage of open surgery. Second, there might also be some inter‐operator variation in surgical treatment of cervical cancer between different oncologic surgeons. Therefore, after controlling for potential risk factors (such as uterine manipulators,^22^ CO_2_ pneumoperitoneum,^23^, ^24^, ^25^ tumor‐free operation) that may cause a poor prognosis of laparoscopic surgery, multicentered, large‐sample, and randomized controlled trials are needed to explore further the oncologic safety and the causes of poor oncologic outcomes for laparoscopic surgery.

## 5 CONCLUSIONS

In conclusion, this study demonstrates the superiority of oncologic outcomes of laparotomy for the treatment of patients with stage IB1‐IB3 cervical cancer. Although the laparoscope approach was associated with less blood loss and shorter length of hospital stay as anticipated, the advantages of open surgery in the benefits of OS and DFS were indeed observed in our study, even in stage IB1 patient with LVSI‐ (2018 FIGO) populations. Currently, we believe that an abdominal surgery approach should be preferred for women with stage IB cervical cancer.

## CONFLICT OF INTEREST

The authors declare no potential conflict of interest.

## AUTHOR CONTRIBUTIONS

Conception and design: Jihong Liu, Danian Dai. Administrative support: Jihong Liu. Collection and assembly of data: Danian Dai, He Huang, Yanling Feng, Ting Wan, Zhimin Liu, Chongjie Tong. Data analysis and interpretation: Danian Dai, He Huang, Yanling Feng, Ting Wan, Zhimin Liu, Chongjie Tong. Manuscript writing: Danian Dai, He Huang, Yanling Feng. Final approval of manuscript: All authors. Accountable for all aspects of the work: All authors.

## Supporting information

Table S1‐S2
