# Supplementary material for: Minimally invasive surgery vs laparotomy for early stage cervical cancer: A propensity score‐matched cohort study
Source: Cancer Med. 2020 Nov 25;9(24):9236–45. doi: 10.1002/cam4.3527 (PMC7774733; doi:10.1002/cam4.3527)
Supplement: Supplementary file 1 — Table S1‐S2 [file CAM4-9-9236-s001.docx]

| **Supplementary Table 1. comparison of operation related indexes between MIS and laparotomy** | | | | |
| --- | --- | --- | --- | --- |
| **Group** | **Case** | **Operative time(min)a** | **Estimated blood loss(mL)a** | **Length of hospital stay(day)a** |
|  |  |  |  |  |
| **MIS** | 213 | 247.7±85.0 | 140.0±150.3 | 6.4±2.7 |
| **laparotomy** | 213 | 212.0±56.2 | 199.1±181.5 | 8.0±2.3 |
| **P-value** |  | **<0.001*** | **<0.001*** | **<0.001*** |
| * p < 0.05, statistically significant.  Using t test, P < 0.05 was considered statistically significant. a Mean±SD Abbreviations: MIS: minimally invasive surgery | | | | |

| **Supplementray table 2. Recurrence and death between MIS and laparotomy group** | | | | |
| --- | --- | --- | --- | --- |
| **Variables** | **Recurrence** | | | **Death** |
|  | **Intra-pelvic** | **Extra-pelvic** | **Total** |  |
| **MIS (n=213)** | 21(9.9%) | 6(2.8%) | 27(12.7%) | 17(8.0%) |
| **laparotomy (n=213)** | 10(4.3%) | 2(0.9%) | 12(5.6%) | 5(2.3%) |
|  |  |  |  |  |
| Abbreviations: MIS: minimally invasive surgery | | | | |
